# Sperm quality and morphometry characterization of cryopreserved canine sperm in ACP-106c or TRIS

**DOI:** 10.1590/1984-3143-AR2021-0069

**Published:** 2022-09-05

**Authors:** Diego Oliveira Teixeira, Herlon Victor Rodrigues Silva, Bruna Farias Brito, Brenna de Sousa Barbosa, Beatriz Evaristo de Almeida Tabosa, Lúcia Daniel Machado da Silva

**Affiliations:** 1 Universidade Estadual do Ceará, Faculdade de Veterinária, Fortaleza, CE, Brasil

**Keywords:** cryopreservation, cryodamage, extenders, morphology, spermatozoa measurement

## Abstract

Morphological sperm evaluation supported by the morphometry can be used in the determination of the seminal quality and in the investigation of potential extenders. Although there are studies comparing TRIS and ACP extenders, there are no comparative studies between them for the computerized assisted semen analysis (CASA), sperm viability, membrane functionality and sperm morphometry parameters of cryopreserved canine semen. Hence, we aimed to evaluate the effects of ACP-106c and TRIS on post-freezing canine sperm quality. Five dogs were submitted to semen collection twice with one-week interval. The semen was evaluated within the parameters: total motility, vigor, concentration, viability, plasma membrane functionality, morphology and morphometry. In the morphometric evaluation, the morphologically normal sperm was measured as: length, width, area and perimeter of the head and the midpiece, tail length and total length. The parameters of ellipticity, elongation, regularity and roughness were determined. Then, the semen was divided into two aliquots that were diluted in TRIS or ACP-106c, with the addition of egg yolk and glycerol. The diluted semen was refrigerated and frozen. The thawed samples were evaluated. Total motility, viability, sperm membrane functionality and normal morphology reduced after thawing in both extenders (morphology reduced from 89.60 ± 1.3% to 84.40 ± 1.8 and 84.60 ± 1.1% in TRIS and ACP-106c, respectively). However, it did not differ between TRIS and ACP-106c. In the ACP-106c the sperm head defects in cryopreserved semen were higher compared to fresh semen (P < 0.05). For all the morphometric parameters evaluated, there were no differences between fresh and cryopreserved samples (3.70 ± 0.4% *vs*. 2.30 ± 0.5%). In kinetics, with an interval of one week statistical differences between the extenders were found only in the parameters ALH and LIN (P < 0.05). Regardless of the extender, there were no changes in the morphometric parameters of sperm after thawing.

## Introduction

Specialized dog breeding has become a highly profitable activity, promoting the improvement of reproductive biotechniques, such as artificial insemination and seminal cryopreservation, aiming at better employ of the genetic material of canine breeders and obtaining offspring with desired breed characteristics and patterns (Rahman et al., 2017).

Semen cryopreservation is advantageous because it protects the genetic potential of males, maintaining the fertilizing capacity of gametes even after the death of the animal. Besides that, it enables the rapid spread of gametes to different regions of the planet (Silva et al., 2018). However, it is emphasized that in the cryopreservation process, gametes can pass through structural and functional changes, such as the rupture of plasma membranes and DNA fragmentation (Sieme et al., 2016). In addition, morphometric changes are also pointed out after cryopreservation process, related mainly to the decrease in the size of the sperm head, resulting from excessive dehydration and loss of the sperm membrane's osmoregulatory mechanisms (Rijsselaere et al., 2004; Hidalgo et al., 2007).

Thus, the morphometric analysis is an essential tool for the observation of cryodamages suffered by gametes, since it offers an objective/standardized assessment of conventional morphology (Valverde et al., 2016; Barbosa et al., 2019). Morphometry allows the detection of subtle differences in the size of the sperm head, undetectable to the morphological evaluation, which interferes in the degree of fertility (Paz et al., 2011), the stability of chromatin (Núñez‐Martinez et al., 2007a) and the crioresistance of the sperm sample (Ramon et al., 2013). In addition, morphometric measurements of the midpiece are related to the swimming movement of the gametes and to the cryodamage to motility (Yániz et al., 2015).

In this context, the morphological evaluation supported by the morphometry can be used in the determination of the seminal quality and in the investigation of potential extenders (Arruda et al., 2002). In the canine species, TRIS-based extenders and powdered coconut water specific for the canine species (ACP-106c) are usually used (Silva et al., 2006; Mota et al., 2014). Powdered coconut water (ACP) is a thinner of vegetable origin, with a rich composition based in proteins, salts, sugars (fructose, glucose and sucrose), vitamins, amino acids and growth factors, which benefits motility and morphology of the sperm after thawing process. On the other hand, the TRIS extender is characterized by the buffering action, promoting the reduction of fructose metabolism in the cell, conserving the gamete motility.

Although there are already studies that have compared these two extenders for canine semen freezing, an evaluation encompassing computerized evaluation, sperm viability, membrane functionality and sperm morphometry had not been performed. There are no comparative studies between the ACP-106c and TRIS extenders for those parameters. Hence, this work aimed to evaluate the effects of cryopreservation on the canine sperm morphometry and quality using ACP-106c and TRIS extenders.

## Methods

### Animals

All procedures performed in this work were approved by the Committee on Ethics in the Use of Animals of the State University of Ceará, number 09943875-2019. The animals used in this experiment came from private kennels.

Five dogs of different breeds were selected, three golden retrievers, one English bulldog and one English cocker spaniel, aged between 2 and 8 years and clinically healthy. They were kept in individual stalls during the experimentation period with *ad libitum* water and fed twice a day with commercial dog food.

### Chemicals

Unless otherwise indicated, all chemicals were purchased from Sigma-Aldrich Chemical Company (St. Louis, MO, USA).

### Preparation of extenders

To carry out the experiment, the TRIS and ACP-106c (ACP Biotechnologia, Fortaleza-CE, Brazil) seminal extenders were used. The ACP-106c extender was prepared according to the manufacturer's recommendations. For this purpose, a package of ACP-106c, containing 12 g of the product, was reconstituted in 50 mL of distilled water, producing a solution of 295 mOsm/L and pH 7.4. To prepare the Tris extender with 305 mOsm/L and pH 6.6, 3.028 g of Tris-hydroxymethyl-aminomethane, 1.78 g of citric acid monohydrate and 1.25 g of D-fructose are dissolved in 100 mL of distilled water (Mota et al., 2014).

The base extenders were added with egg yolk (20% for TRIS and 10% for ACP-106c) and 6% glycerol (Mota et al., 2014) as the cryoprotectants, composing the cryopreservation media.

### Semen collection, freezing and thawing

Semen collection was performed by digital manipulation. Each dog was submitted to two collections, in a period of one week between them, resulting in ten semen samples. The ejaculate obtained was evaluated and only samples with total motility> 80% were used in the experiment, as recommended by CBRA (2013).

After approximately 2 minutes of obtaining the ejaculate, the sample was fractionated. The ejaculate was fractionated in two 15 ml Falcons tubes, preheated to 37 ºC, then diluted in the TRIS and ACP-106c extenders, obtaining a final sperm concentration of 150 x 10^6^ sptz/mL. Next, the samples were stored in a thermal box with recyclable ice (15 °C) for approximately 40 minutes. Subsequently, the sperm samples were directed to a refrigerator (4 ºC) for 30 minutes. After, the samples were packaged in French straws of 0.25 mL and placed horizontally on a freezing ramp at a height of 5 cm from the liquid nitrogen slide, for 5 minutes. The straws containing the samples were stored in cryogenic cylinders (Silva et al., 2018).

The thawing process was carried out after a minimum of 7 days of freezing. The protocol consisted of removing the frozen straws from the cylinder and immersing them in a water bath programmed at 37 ºC for 1 minute (Silva et al., 2018). The sperm samples were transferred to 2 mL plastic tubes, heated to 37 ºC and, immediately submitted to sperm evaluation.

### Sperm evaluation

The total motility (percentage of cells in motion) and the vigor (quality of progressive sperm movement on a scale from 0 to 5) parameters were evaluated using10 μL of the sperm sample placed on a pre-heated slide (37 ºC), with observations of three different fields of the slide, under an optical microscope (100x) (CBRA, 2013).

The sperm concentration was determined by diluting 5 μL of the biological sample with 95 μL of 0.1% formal-saline solution (1:10). The counting proceeded in a Neubauer chamber with the use of a microscope (400x) (Cardoso et al., 2006).

The quantification of viability was performed by the bromophenol blue as vital stain. In a preheated slide (37 ºC), 10 μL of the sperm sample were added in 5 μL of the dye, followed by the smear and counting of 100 random cells, between viable (discolored or transparent) and non-viable (colored or blue), under an optical microscope (400x) (Costa et al., 2013).

The membrane functionality was evaluated using the hypoosmotic test. For this purpose, a 10 μL aliquot of the semen was incubated in 90 μL of hypoosmotic solution (300 mOsm/L), for 45 minutes at 37 ºC. Then, a random count of 100 sperm under a microscope (400x) was performed. The sperm were then classified into: functional membrane (swollen and coiled tail sperm), and non-functional membrane (no reaction - straight tail) (Cardoso et al., 2006).

For the morphological evaluation, a smear was made from 5 μL of the sample diluted in 45 μL rose Bengal 1.5% stain solution. A total of 100 sperm were randomly counted under a microscope (1000x) and classified as normal or abnormal. Sperm abnormalities were grouped into changes in the head, midpiece, tail and acrosome (CBRA, 2013; Barbosa et al., 2019).

The morphometry was performed by capturing 100 images of sperm previously analyzed and considered morphologically normal, on slides stained with rose bengal, using the Nikon Eclipse 80i phase contrast microscope (Nikon, Tokyo, Japan) coupled to the Control Unit DS-U2 camera (Control Unit DS-U2 camera) and NIS-Elements AR3.2 software (Nikon, Tokyo, Japan). The images were obtained at 1000x magnification. The final resolution of the images was 1280 x 1024 pixels. Measurements were performed using ImageJ software version 1.4.3.67. Measurements with ImageJ followed the recommendations of published works that used this software for sperm morphometric characterization (Tremori et al., 2014; Barbosa et al., 2019). Measurements were performed by manually clicking on the image, selecting the appropriate software tool for the width and length dimensions (line selection tools), and for area and perimeter (area selection tools). Regarding the limit, the software was previously calibrated using the scale offered in the image to be analyzed. This scale is generated by the capture software, NIS-Elements AR3,2. For each cell it was measured: length, width, area and perimeter of the head; length, width, area and perimeter of the midpiece; tail length and total length of the sperm. From the data obtained from the measurements of the sperm, head, ellipticity, elongation, roughness and regularity were calculated according to Barbosa et al. (2019).

The evaluation of sperm kinetics was performed on the thawed sample by Computerized Assisted Semen Analysis (CASA), software Sperm Class Analyzer® version 5.3.0.1 (SCA®, Microptic SL, Barcelona, Spain). CASA was not used to evaluate fresh samples because the dogs were far from the laboratory where CASA is located. Therefore, CASA was used only to evaluate post-thawing samples. For this, a 10 μL aliquot of each sample was placed in a Makler Chamber, previously heated (37 ºC) and evaluated in a phase contrast microscope coupled with a digital camera (Costa et al., 2013). The samples were evaluated in three randomly selected, nonconsecutive microscopic fields. The fields that presented a better visualization of the cells were used for the evaluation, allowing the recognition by the software. Edits were performed only to remove egg yolk clumps that could interfere with the analysis result. The settings were: frame rate, 25 frames/s; minimum contrast, 75; straightness threshold, 75%; low velocity average pathway (VAP) cutoff, 10; and medium VAP cutoff, 55. The parameters evaluated were: total motility - TM (%), progressive motility (%),VAP (μm/s), velocity straight line - VSL (μm/s), velocity curvilinear - VCL (μm/s), amplitude of lateral head displacement - ALH (μm), beat cross frequency - BCF (Hz), straightness - STR (%), linearity - LIN (%), wobble coefficient (WOB) (Mota et al., 2014).

### Statistics

The results were expressed as mean ± standard error. The data were analyzed using the statistical software R-project © version 3.3.2 (The R Foundation, Vienna, Austria). They were initially submitted to Shapiro Wilk and Levene’s tests for normality and homoscedasticity, respectively. The comparison of fresh semen with cryopreserved samples, as well as between extenders, TRIS and ACP-106c, was performed by the T test for the parametric data and the Mann-Whitney test for the non-parametric ones. The results were considered significant when P < 0.05.

## Results

The initial quality of the ejaculate showed means of 94.00 ± 7.1% and 4.7 ± 0,6 for total motility and vigor. The mean initial sperm concentration of ejaculates was 319.8 x 10^6^ sptz/mL.

The viability, membrane functionality and fresh semen morphology (Table 1) were compared to the cryopreserved samples (P < 0.05). However, no differences were observed between those samples with TRIS and ACP-106c (P > 0.05). The reduction in the percentage of normal sperm after cryopreservation (from 89.60 ± 1.3% to 84.40 ± 1.8 and 84.60 ± 1.1% in the TRIS and ACP-106c groups, respectively) was followed by significant increase in sperm head defects in the ACP-106c group (3.70 ± 0.4%) compared to the fresh control (2.30 ± 0.5%) (P < 0.05).

**Table 1:** Viability, plasma membrane functionality and morphology (mean ± SE) of fresh and cryopreserved canine sperm in TRIS and ACP-106c extenders.

**Parameters**	**Fresh**	**Thawed**	
		**TRIS**	**ACP-106c**
Viability (%)	91.80 ± 1.6 ^a^	73.30 ± 4.0 ^b^	63.30 ± 4.3 ^b^
Functional membrane (%)	92.80 ± 1.5 ^a^	73.60 ± 3.3 ^b^	63.90 ± 4.6 ^b^
Normal morphology (%)	89.60 ± 1.3 ^a^	84.40 ± 1.8 ^b^	84.60 ± 1.1 ^b^
Acrosome defect (%)	0.00 ± 0.0 ^a^	0.70 ± 0.4 ^a^	0.20 ± 0.1 ^a^
Head defect (%)	2.30 ± 0.5 ^a^	2.50 ± 0.7 ^ab^	3.7 ± 0.4 ^b^
Midpiece defect (%)	0.30 ± 0.2 ^a^	0.80 ± 0.4 ^a^	0.90 ± 0.6 ^a^
Tail defect (%)	7.80 ± 1.0 ^a^	11.70 ± 1.7 ^a^	10.60 ± 0.9 ^a^

Different lower case letters on the same line means statistical difference between groups (P < 0.05).

The data regarding the morphometric measurements of cryopreserved canine sperm in TRIS and ACP-106c are shown in Table 2. For all morphometric measurements evaluated, no statistical differences were observed between the fresh and the cryopreserved samples, regardless of the extender used in the experiments (P > 0.05).

**Table 2:** Morphometry (mean ± SE) of fresh and cryopreserved canine sperm (sptz) in TRIS and ACP-106c extenders.

**Parameters**	**Fresh**	**Thawed**	
		**TRIS**	**ACP-106c**
**Head**			
Length	6.80 ± 0.9 ^a^	6.73 ± 0.09 ^a^	6.76 ± 0.9 ^a^
Width	4.32 ± 0.10 ^a^	4.20 ± 0.08 ^a^	4.30 ± 0.11 ^a^
Area	26.29 ± 0.82 ^a^	25.31 ± 0.74 ^a^	25.91 ± 0.93 ^a^
Perimeter	19.99 ± 0.28 ^a^	19.69 ± 0.28 ^a^	19.90 ± 0.36 ^a^
Ellipticity	1.59 ± 0.03 ^a^	1.61 ± 0.03 ^a^	1.59 ± 0.04 ^a^
Elongation	27.67 ± 1.08 ^a^	27.24 ± 1.04 ^a^	27.70 ± 1.19 ^a^
Rugosity	0.82 ± 0.01 ^a^	0.82 ±0.01 ^a^	0.82 ± 0.01 ^a^
Regularity	3.54 ± 0.07 ^a^	3.53 ± 0.04 ^a^	3.55 ± 0.05 ^a^
**Midpiece**			
Length	10.93 ± 0.17 ^a^	11.05 ± 0.14 ^a^	10.89 ± 0.15 ^a^
Width	1.05 ± 0.05 ^a^	1.07 ± 0.06 ^a^	1.05 ± 0.04 ^a^
Area	13.80 ± 0.68 ^a^	13.08 ± 0.63 ^a^	13.01 ± 0.69 ^a^
Perimeter	24.86 ± 0.16 ^a^	24.69 ± 0.24 ^a^	24.56 ± 0.20 ^a^
Tail length	45.49 ± 0.43 ^a^	45.03 ± 0.31 ^a^	44.42 ± 0.40 ^a^
**Total length of sptz**	63.23 ± 0.50 ^a^	62.81 ± 0.42 ^a^	62.08 ± 0.48 ^a^

Different lower case letters on the same line means statistical difference between groups (P < 0.05).

Almost all kinetic parameters of frozen semen were similar between TRIS and ACP-106c extenders (P > 0.05), with difference only in ALA and LIN. The ALH of the TRIS group (3.66 ± 0.2 μm) was significantly higher than the ACP-106c (2.91 ± 0.2 μm), whereas the LIN of theACP-106c (73.11 ± 2.6) was higher than TRIS (73.11 ± 2.6 – Table 3).

**Table 3:** Kinect parameters (mean ± SE) of cryopreserved canine sperm in TRIS e ACP-106c extenders analyzed by computerized assisted semen analysis.

**Parameters**	**TRIS**	**ACP-106c**
Total motility (%)	47.95 ± 7.0 ^a^	42.09 ± 7.0 ^a^
Progressive motility (%)	10.81 ± 2.2 ^a^	8.70 ± 2.6 ^a^
Average path velocity (µm/s)	67.15 ± 3.5 ^a^	68.38 ± 3.7 ^a^
Straight line velocity (µm/s)	60.90 ± 3.6 ^a^	63.24 ± 3.9 ^a^
Curvilinear velocity (µm/s)	88.81 ± 5.1^a^	86.61 ± 4.5 ^a^
Amplitude of lateral head displacement (µm)	3.66 ± 0.2 ^a^	2.91 ± 0.2 ^b^
Beat cross frequency (Hz)	11. 53 ± 0.5 ^a^	11.23 ± 0.6 ^a^
Straightness (%)	90.42 ± 1.0 ^a^	92.16 ± 1.2 ^a^
Linearity (%)	68.68 ± 1.0 ^b^	73.11 ± 2.6 ^a^
Wobble coefficient (%)	75.97 ± 1.1^a^	79.15 ± 2.1 ^a^

Different lower case letters on the same line means a statistical difference between post-thaw treatments (P < 0.05).

## Discussion

The choice of the appropriate extender for sperm characteristics is essential for the success of gamete cryopreservation. In this work, TRIS and ACP-106c showed no differences for the main parameters indicating sperm quality, being in agreement with previous studies (Silva et al., 2006; Mota et al., 2014; Barbosa et al., 2019). These results support the effectiveness of both extenders in the conservation of gametes, giving the researcher in charge the option of choosing the medium that presents requirements in addition to efficiency, such as cost, availability and practicality.

Regarding the morphology, the reduction in the percentage of normal post-cryopreservation sperm is expected given the changes in osmolarity and temperature at which these cells are subjected (Rijsselaere et al., 2004; Barbosa et al., 2019). Some authors mention that the most critical steps to these changes are dilution, freezing and thawing (Costa et al., 2013). In addition, they show the prevalence of tail defects in cryopreserved sperm, followed by head and midpiece defects (Cardoso et al., 2003, 2006; Barbosa et al., 2019). It corroborates with the findings of this study, in which a greater proportion of changes in the sperm tail was observed in relation to the other defects within the group of fresh and cryopreserved samples.

The percentage of normal sperm after thawing did not differ between the TRIS and ACP-106c treatments, similar to the data documented by Mota et al. (2014). Both diluters tested are within the values accepted in the literature, which describe a 10 to 20% decline in sperm morphology for cryopreserved samples in relation to fresh semen (Robert et al., 2016). The morphological data obtained in the study remained close to 84%, indicating that the extenders were able to ensure the morphological integrity of the cell, without causing significant losses to the parameter.

A significant increase in head defects was observed in cryopreserved sperm with the ACP-106c extender. Possibly, it is due to a greater interference of the components of the ACP-106c extender in the process of dehydration and rehydration during the freezing curve (Oliveira et al., 2011). It is known that ACP-106c is rich in sugars such as glucose, fructose and sucrose, which can lead to the appearance of a hypertonic extracellular environment that may further aggravate stress (Cardoso et al., 2003).

The quantification of acrosome defects did not increase after cryopreservation. It is believed that the presence and/or supplementation of the media with sucrose and other monosaccharides, especially fructose, protects the acrosome from possible cryodamages, contributing to the integrity of this post-thaw structure (Yildiz et al., 2000). In addition, maintaining the normal sperm tail morphology is an indication that both extenders provided an adequate osmotic environment and that the freeze-thaw process was compatible with canine sperm sensitivity (Arruda et al., 2015).

Regarding morphometry, the measurements of the sperm head (length, width, area and perimeter) of this study are close to the values reported in the canine specie (Rijsselaere et al., 2004; Núñez‐Martinez et al., 2005, 2007b; Soler et al., 2017). Differences in morphometric data among studies are the result of different methods and tools for measurement / evaluation. Most of the studies cited used computer systems to obtain sperm morphometry, these being CASA-Morph, CASMA or Metrix Oval Head Morphology implemented in Hamilton-Thorne CEROS (Arruda et al., 2002; Cardoso et al., 2006; Silva et al., 2006; Costa et al., 2013; Mota et al., 2014; Yániz et al., 2015). This work opted to use the ImagJ tool in morphometric measurement due to its accessibility and low cost. This methodology allows the generation of reliable data and it has already been used in the morphometric characterization of sperm of different species (Silva et al., 2015; Barbosa et al., 2019; Cunha et al., 2021).

Morphometry is also influenced by the cryopreservation process, by the type of extender, by the species and/or breed of the animal (Chirinea et al., 2013; Soler et al., 2017). In this work, however, the sperm did not differ between the tested extenders or between the evaluation times, for any of the measured parameters. This indicates the effectiveness of the TRIS and ACP-106c extenders in maintaining the integrity of the dimensions of the cryopreserved gamete structures.

The effects of cryopreservation on sperm head morphometry have been reported in human (Maree et al., 2010), bovine (Valverde et al., 2016), ovine (Ramon et al., 2013), caprine (Hidalgo et al., 2007), equine (Arruda et al., 2002), canine (Silva et al., 2018) species and in wild boars (García‐Herreros et al., 2008). All of these studies have shown that the sperm submitted to cryopreservation undergoes a significant decrease in the morphometric dimensions of the head, which are justified by possible damage or loss of the acrosome (Rijsselaere et al., 2004; Núñez‐Martinez et al., 2007b); super condensation of chromatin (Núñez‐Martinez et al., 2007b) and osmotic changes associated with excessive cell dehydration (Arruda et al., 2002).

Gravance et al. (1997), however, demonstrated that cryopreserved goat sperm did not reduce the dimensions of the sperm head. These conflicting data is probably due to species-specific crioresistance and to the freezing protocol, aspects such as glycerol concentration and refrigeration curve, resulting in different effects on post-thaw sperm characteristics (Salazar et al., 2011). Recent work with canine sperm also showed similarity between the results of length, width, perimeter and head area of the fresh sample, which passed through slow freezing. In this case, it was reported that the vitrification of the gametes changes the post-heating morphometric dimensions, with differences observed between the measurement for the two proposed cryopreservation techniques (Cerdeira et al., 2020). Accordingly, we hypothesized that good quality semen (as used in our study) submitted to the appropriate cryopreservation methodology minimizes cryogenic damage to sperm and, consequently, the effect of cryopreservation on head morphometry is smaller, conserving the dimensions close to that in fresh semen.

Regarding the morphometry of the midpiece, it is important in the correlation between motility and energy production of the gamete. Studies point to an intimate relationship between the volume of the midpiece and the crioresistance, since the mitochondria, abundantly present in this region, are extremely sensitive to freeze-thaw (CBRA, 2013; Figueroa et al., 2019). We chose to measure width, area, length and perimeter aiming to create reference data for future investigations on sperm shape/function in dogs, where such data are lacking.

The width and the area of the midpiece evaluated in this experiment were greater when compared to the data obtained by Núñez‐Martinez et al. (2005) for canine specie. The intervals observed in these measurements are between 0.56 and 1.7 µm for width and 8.01 and 27.97 µm for area in fresh and cryopreserved samples. To the present date, the publication of other works that expose morphometric data for the region of the midpiece in canine gametes is non-existent. This highlights the importance of this study in creating a morphometric database for canine sperm.

The average length of the sperm tail varied between 39.97μm to 62.02μm, considering fresh and cryopreserved samples. Even without showing a statistical distinction, there was a tendency to reduce the length of the post-thaw flagellum, especially in the ACP-106c group. This possible effect is due to the dehydration and hydration mechanisms to which the cell is subjected during the refrigeration and thawing steps, respectively. This mechanism can be enhanced according to the extender composition (Sieme et al., 2016).

The lack of morphometric data regarding the flagellum portion of the canine gamete was also observed. In a previous study, researchers demonstrated a significant reduction in the sperm flagellum in cryopreserved samples when compared to fresh samples (Rijsselaere et al., 2004). The flagellum presents great fragility of its internal and external structures when exposed to the cryopreservation process. A study with red deer correlates the flagellum size to positive results in semen cryopreservation (Ros-Santaella et al., 2014). The role of the flagellum as a modulator of sperm function, displacement velocity and fertility potential and an indicator of crioresistance should be explored in future morphometric studies.

The description of the ellipticity, elongation, roughness and regularity of the sperm head help in the objective distinction of the normal gamete shape (Barbosa et al., 2019). The data expressed in this study are in agreement with what has already been reported; although some variations are noticed due to the difference of the breeds (Soler et al., 2017; Urbano et al., 2017]. The shape of the sperm is a determining factor for reproductive success. The literature demonstrates the relationship between high DNA denaturation and the flattened sperm head pattern in fresh canine semen (Núñez‐Martinez et al., 2007a). Crioresistance pattern was also observed in small, elongated and narrow head sperm in red deer (Esteso et al., 2006). Understanding these variables helps in the development of a cluster to select subpopulations that have greater resistance to cryopreservation and/or predilection for a specific extender (CBRA, 2013; Yániz et al., 2015).

Finally, regarding CASA results, the values recorded for motility and kinetic velocity parameters are equivalent to those obtained in previous studies, both for TRIS and ACP-106c extenders (Uchoa et al., 2012; Costa et al., 2013). The ACP-106c group showed lower ALH than TRIS, which is not good regarding the functionality of the gamete, since this parameter is closely related to the ability of the sperm to penetrate in vivo and in vitro and to the gamete hyperactivation process (Cardoso et al., 2006; Barbosa et al., 2020). Another divergent kinetic variable among the evaluated extenders was linearity, which was higher in the ACP-106c group. Linearity is given as a function of the relationship between VSL and VCL; it infers in the migration and penetration of the sperm in the female's cervical mucus (Matos et al., 2008).

## Conclusion

In conclusion, the morphometric measurement demonstrated the efficiency of the protocol in preserving the dimensions of the canine gamete. In sum, the lack of comparative pre- and post-freezing morphometric studies, considering the use of different cryoprotective media and the choice of the variables investigated here confirm the importance of the present study in the creation of a canine sperm morphometric database that can be used as a support for further functional, structural and evolutionary investigations of gametes.
